# Spontaneous Loculated Pneumomediastinum in a COVID-19-Infected Patient

**DOI:** 10.1155/2022/5943221

**Published:** 2022-05-30

**Authors:** Sirous Jafari, Zahra Jahani, Reihane Alikhani, SeyedAhmad SeyedAlinaghi, Malihe Hasannezhad, Faeze Salahshour, Ali Asadollahi-Amin

**Affiliations:** ^1^Department of Infectious Diseases, Imam Khomeini Hospital Complex, Tehran University of Medical Sciences, Tehran, Iran; ^2^Iranian Research Center for HIV/AIDS, Iranian Institute for Reduction of High-Risk Behaviors, Tehran University of Medical Sciences, Tehran, Iran; ^3^Department of Radiology, Advanced Diagnostic and Interventional Radiology Research Center (ADIR), Tehran University of Medical Sciences, Tehran, Iran

## Abstract

While we are still learning about COVID-19 affecting people, older persons and persons with underlying diseases such as high blood pressure, heart disease, and diabetes mellitus (DM) appear to develop serious illness and more complications often than others. In this report, we presented a patient with spontaneous pneumomediastinum after COVID-19. The patient was a 61-year-old man with a history of DM, hypertension, and heart failure, who has been infected with COVID-19. The patient was diagnosed with COVID-19 based on RT-PCR analysis of nasopharyngeal samples, and chest X-ray showed patchy infiltration upper and lower lobes bilaterally. By day 4, imaging was repeated, performed due to exacerbation of pleuritic chest pain, decreased O_2_ saturation (80%), and coughing that revealed multiple ground-glass opacities bilaterally, and interlobular septal thickening with emphysema in most of the left upper lobe and a small part of right upper lobe which led to severe spontaneous left pneumomediastinum and parenchymal consolidation was also observed. The combination of a chest tube, antibiotics (vancomycin 1 gr/bid and meropenem 1 g/bid), and antiviral (hydroxychloroquine 200 mg/bid and atazanavir 300 mg/daily) was prescribed, and continued treatment with antiviral and appropriate care for pneumomediastinum was successful. Spontaneous pneumomediastinum in the context of COVID-19 should be considered as a prognostic factor in favor of worsening diseases.

## 1. Introduction

Human coronavirus is one of the main pathogens of respiratory infection. The two highly pathogenic viruses, SARS-CoV and MERS-CoV, cause a severe respiratory syndrome in humans, and four other human coronaviruses (HCoV-OC43, HCoV-229E, HCoV-NL63, and HCoV-HKU1) induce mild upper respiratory disease. Most patients have mild symptoms and a good prognosis [1].

So far, a few patients with 2019-nCoV have developed severe pneumonia, pulmonary edema, acute respiratory distress syndrome (ARDS), or multiple organ failure and have died. The 2019-nCoV infection was of clustering onset, is more likely to affect older men with comorbidities, and could result in severe and even fatal respiratory diseases such as ARDS. Early identification and timely treatment of critical cases of 2019-nCoV are important. Effective life support and active treatment of complications should be provided to effectively reduce the severity of patients' conditions and prevent the spread of this new coronavirus worldwide. Among the many unusual findings, some involve the pulmonary system; however, pneumomediastinum has rarely been reported [2–6]. The purpose of this report was to describe the rare complication in a patient with COVID-19.

## 2. Case Presentation

A 61-year-old man with a history of diabetes mellitus (DM), hypertension, and heart failure from Amol, Iran, was admitted to the Amol Imam Khomeini Hospital, on April 07, 2020, with 7 days history of fever, dyspnea, severe nonproductive cough, and pleuritic chest pain. On admission, in physical examination (P/E), the patient was febrile (38°C) with 92% O_2_ saturation. Complete blood count (CBC) showed elevated leukocytes (38,100 cells per *μ*L (normal range 4,100–10,100 cells per *μ*L)) and neutrophils (8,382 cells per *μ*L (1,800–6,300 cells per *μ*L)), while the lymphocyte count (27,813 cells per *μ*L) was in the normal range (1,100–3,200 cells per *μ*L). Laboratory prognostic tests showed an increase level of C-reactive protein (CRP) 15 mg/L (adult < 10 mg/L), LDH 659 unit/L (<480 unit/L), D-dimer 838 ng/ml (>1,000 ng/ml), troponin < 1.5 ng/L (<100 ng/L), and CPK 108 U/L (55–170 U/L). More importantly, chest X-ray (CXR) showed patchy infiltration upper and lower lobes bilaterally.

**Figure 1:**
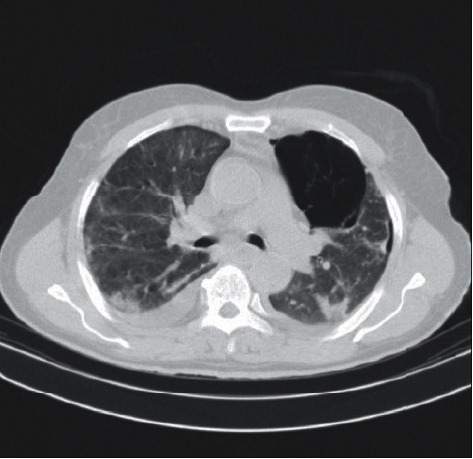
Chest CT showing patchy peripheral ground-glass opacities, and scattered subsegmental atelectatic bands are compatible with COVID-19 pneumonia. A small amount of pneumothorax is seen in the right hemithorax particularly anteroinferiorly. Large emphysematous bulla is seen in the medial aspect of the left upper lobe, and a smaller similar bulla is seen in the right paracardiac region. Mild bilateral pleural effusion is also evident.

The patient was given vancomycin 1 g/bid and meropenem 1 g/bid for 13 days and hydroxychloroquine (HCQ) 200 mg/bid for 2 days, and HCQ was ceased, while Kaletra was initiated, Kaletra 400/100 mg bid for 11 days and vitamins C and B complex for 6 days. After 4 days, the patient was diagnosed with coronavirus disease 2019 (COVID-19) based on RT-PCR analysis of nasopharyngeal samples. According to exacerbation of pleuritic chest pain, decreased O_2_ saturation (80%), and coughing, imaging was repeated. It revealed multiple ground-glass opacities bilaterally and interlobular septal thickening with emphysema in most of the left upper lobe and a small part of right upper lobe which led to severe spontaneous left loculated pneumomediastinum and parenchymal consolidation were also observed (Figure 1). Eventually, the patient underwent a chest tube insertion in left anterior axillary and transferred to the intensive care unit (ICU), and the supplemental oxygen, antibiotics, antiviral, and the other medical treatment were continued.

**Figure 2:**
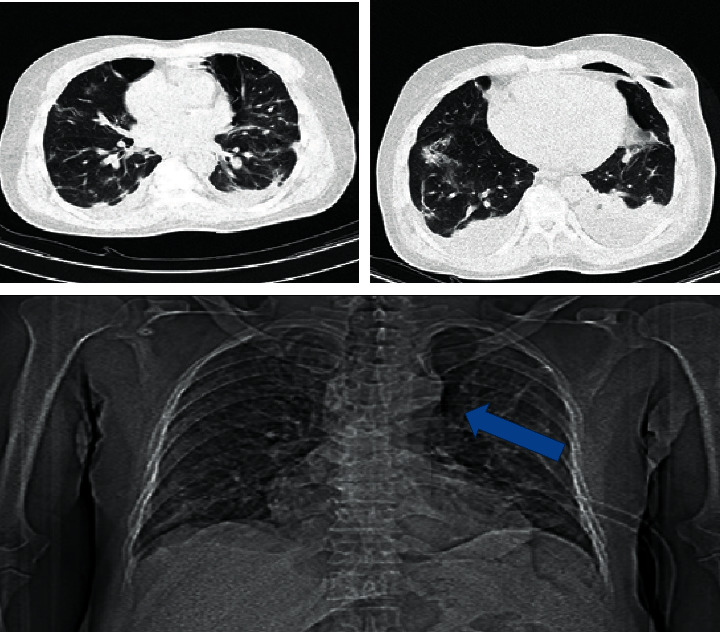
Chest CT showing spontaneous pneumomediastinum (arrow), emphysema, bilateral pleural effusion, and ground-glass opacities of the lung, which decreased after treatments.

By day 11, the chest tube came out following improved shortness of breath, pleuritic chest pain, and coughing, as well as O_2_ saturation (90%). Another CT scan was required for further evaluation which showed significantly improved left emphysema, but bilateral mild to moderate pleural effusion has been achieved with the reduction of pneumomediastinum and parenchymal consolidation (Figure 2).

By day 13, the patient was referred to the Imam Khomeini Hospital Complex affiliated to Tehran University of Medical Sciences, Tertiary Care Hospital, on April 21, 2020. He presented with fever (temperature was 37.9°C), dyspnea, severe nonproductive cough, and pleuritic pain and continued in the medical center. Therefore, the patient was asked for reimaging. Interestingly, CT scan continued to show pneumomediastinum, but more than day 11, on both the sides and even more interesting, the mild to moderate bilateral pleural effusion was completely resolved after 2 days. Due to the lack of complete recovery in the previous hospitalization, treatment continued with HCQ 200 mg/bid, and atazanavir 300 mg/daily was added to his regimen. By day 15, the patient was afebrile (36.8°C). The shortness of breath, chest pain, and cough resolved entirely, and the repeated RT-PCR was negative, and the patient was discharged. We monitored the patient's pneumomediastinum closely according to the consultation with a surgeon based on normal oxygen saturation and stable vital signs. Finally, due to the clinical improvement of the respiratory condition and no chest pain, the follow-up stopped after one month.

## 3. Discussion

Known typical features of COVID-19 on initial CT are bilateral multilobe ground-glass pacifications with a peripheral distribution. Based on currently available information and clinical expertise, spontaneous pneumomediastinum is a rare complication of COVID-19 [3, 7]. In one study, there was only one case of pneumothorax among 99 patients with COVID-19 [1]. Acute deterioration with rapid oxygen desaturation in a COVID-19 patient could indicate pneumothorax, pneumomediastinum, or acute respiratory distress syndrome (ARDS) [7–9].

Although the exact mechanism is unknown, spontaneous loculated pneumomediastinum is usually a self-limiting disease. Several risk factors such as tobacco smoking, male sex, tall and thin stature, non-Hispanic white race, atmospheric pressure changes, and some diseases such as chronic obstructive pulmonary disease (COPD) play a role in spontaneous pneumothorax [10].

Spontaneous rupture of a subpleural bulla is the most likely cause of primary spontaneous pneumothorax [11]. Spontaneous pneumomediastinum refers to alveolar rupture due to an increase in intrathoracic pressure, followed by air dissection through the bronchovascular sheath into the mediastinum. Spontaneous pneumomediastinum can potentially cause severe circulatory and respiratory pathology.

Given that the patient presented at the beginning of the COVID-19 pandemic, our limitations were on the medication and lack of knowledge about the length of the follow-up period and its methods. The best management of such cases remains unknown. However, spontaneous loculated pneumomediastinum usually is a self-limiting disease or gradually improves with long-term oxygen therapy, but sometimes, due to a respiratory disorder and a clear drop in blood oxygen, an invasive intervention such as a chest tube may be needed, like our patient. Steroid therapy as a modification of a cytokine storm in COVID-19 infection can be used and may improve the course of the disease [7]. However, the benefits to its detriment should be weighed. The critical point is that spontaneous pneumomediastinum may occur in the context of COVID-19 as a prognostic factor in favor of worsening disease. However, our approach showed that the management of spontaneous pneumomediastinum due to COVID-19 may be similar to the other conditions.

## Data Availability

The data used to support this study are available from the corresponding authors upon request.
